# A Microscope Automated Fluidic System to Study Bacterial Processes in Real Time

**DOI:** 10.1371/journal.pone.0007282

**Published:** 2009-09-30

**Authors:** Adrien Ducret, Etienne Maisonneuve, Philippe Notareschi, Alain Grossi, Tâm Mignot, Sam Dukan

**Affiliations:** Aix Marseille Université - Laboratoire de Chimie Bactérienne (UPR 9043) - Institut de Microbiologie de la Méditerranée (IFR 88) - CNRS, 31, Chemin Joseph Aiguier, Marseille, France; Baylor College of Medicine, United States of America

## Abstract

Most time lapse microscopy experiments studying bacterial processes *ie* growth, progression through the cell cycle and motility have been performed on thin nutrient agar pads. An important limitation of this approach is that dynamic perturbations of the experimental conditions cannot be easily performed. In eukaryotic cell biology, fluidic approaches have been largely used to study the impact of rapid environmental perturbations on live cells and in real time. However, all these approaches are not easily applicable to bacterial cells because the substrata are in all cases specific and also because microfluidics nanotechnology requires a complex lithography for the study of micrometer sized bacterial cells. In fact, in many cases agar is the experimental solid substratum on which bacteria can move or even grow. For these reasons, we designed a novel hybrid micro fluidic device that combines a thin agar pad and a custom flow chamber. By studying several examples, we show that this system allows real time analysis of a broad array of biological processes such as growth, development and motility. Thus, the flow chamber system will be an essential tool to study any process that take place on an agar surface at the single cell level.

## Introduction

In bacteria, population heterogeneity is somewhat of a novel concept: for a long time, it was considered that cells engaged in a specific physiological phase, for example the exponential or the stationary phase, were all synchronous and equivalent. Such conception is largely the result of the experimental methodology that has employed bulk population-based assays to study bacterial physiology. In other words, cell behaviours in response to various stimuli or environmental changes have largely been inferred from observations obtained at the population level [1], [2]. A wealth of information has been obtained from these studies but the recent emergence of single cell analysis technology has shown that heterogeneity within a phase-synchronized bacterial population is not negligible [3]–[5]. Data is accumulating to show that cellular subtypes have major contributions to global bacterial physiology; striking examples have included the phenomenon of bi-stability, distinct sub cells within single species biofilms and asymmetric aging [4], [6]. In fact, heterogeneity may be the hallmark of most bacterial molecular processes: model exponential phase promoters fused to *gfp*, encoding Green Fluorescent Protein (GFP), did not express equivalent levels of GFP in all cells but, instead, showed a broad array of fluorescence intensities [7].

Studying heterogeneity requires methods allowing the estimation and the quantitation of individual cells within a population [8]. Flow cytometry is a method of choice because it enables the analysis of single cells within a large population, directly in suspension [9], [10]. Cytometry is a powerful quantitative method: analysis of very large cell numbers allow the description of rare phenomena and validation with sound statistics. A limitation of cytometry is that it scans a cellular state at a given time and cannot easily determine the fate of a cell type of interest. This limitation is overcome by microscopy which allows quantitative analysis of biological specimens in time and space and remains the main way to monitor the fate of cells. Microscopy has been widely used to study essential processes such as cell division, sporulation, motility and cell death [3], [11]–[14]. Until now, most time lapse microscopy experiments of bacterial processes *ie* growth, cell cycle and motility studies have used thin agar pads to provide a substratum that supports growth, starvation or surface motility [5], [12], [15], [16]. This method is extremely convenient and is now used standardly in laboratories that study bacterial physiology and cell biology. Unfortunately, the agar pad technique has two major limitations: (i) it is impossible to change the growth conditions by switching the medium composition and study the reversible effect of drugs; (ii) desiccation may also occur and lead to stress, hampering studies over the course of several hours.

These limitations have motivated the development of fluidic techniques to study the behaviour of cells following environmental perturbations, in real time. In eucaryotic cell biology, microscale flow networks have been used to investigate essential processes such as mammalian cell migration [17]–[19], human cell differentiation [20] or even *C. elegans* neuronal activity [21]. However, this nanotechnology is difficult to apply for small bacterial cells: the study of bacteria or even yeast require complex lithography because the set up must be designed to constrain single cells spatially [3], [22]. It is also required that cells be attached to the substratum to resist the flow [23]. Bacterial cells may be functionalized to microfluidic chambers but these approaches are not suitable to all applications: after cell division, daughter cells are unattached and lost in the flow, hampering lineage studies; adhesion or motility studies often require specific substrata which cannot be used easily in microfluidic devices.

Because of these problems and the fact that the agar pad is almost a “universal” substratum in microbiology, we aimed to develop a novel hydrid micro fluidic device that combines a thin agar pad and a custom flow chamber. Most bacterial cells are detached by the flow when simply laid on agar; to overcome this problem, cells were insulated from the flow so that molecules reached the cells by diffusion through the thin agar pad. Here, we provide evidence that diffusion readily occurs in the system and allows for reversible experiments. We further show how the system may be used to study the formation of bacterial micro-colonies and surface motility. Our system is simple and amenable to standard microscopy technology, it is designed for a broad range of applications in bacteriology.

## Results

### Design of the flow chamber device

**Figure 1:**
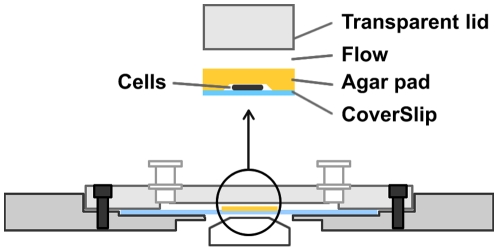
Layout of the microfluidic device. Cells are confined between the coverslip and a 0.5 mm thin layer of agar. The chamber is sealed with a transparent lid containing two entries allowing flow injections. Injected molecules reach the biological specimen by diffusion through the thin layer of agar.

Most commercial uncoated chambers are not suitable for bacteria because the cells resuspend readily when flows are applied. Also, standard coatings are not suitable because the cells bind poorly or binding is toxic (such as poly-lysine substrates). In bacteria, most time-lapse experiments have been performed on agar, so we decided to develop a chamber that would use agar as the main substrate. On agar pads, bacterial cells such as *E. coli* cells bind non-specifically and divide with expected generation times over several hours [5], [12]. However, bacterial cells resuspend very rapidly when liquid is added atop the pad even in the absence of flow (data not shown). To overcome this problem we designed a chamber in which the cells are physically isolated from the liquid flow by the agar pad itself. Schematically, the cells are placed immediately on a coverslip and overlaid with a 0.5 mm thin layer of agar and left to dry gently to absorb the cells onto the agar substrate (Fig. 1). The chamber is then sealed with a transparent lid containing two entries allowing for flow injections (Fig. 1 & Fig. S1A). Flow injections are performed with home-developed computerized electronic injectors, allowing injection of flows ranging between 0,1–100 µL/s. Even at maximum flow rates, injections did not significantly detach the agar pad from the coverslip. Thus, in this chamber system, injected molecules reach the biological specimen by diffusion through the thin layer. In our system up to 12 different solutions can be injected sequentially in the course of the time-lapse experiment (Fig. S1B). The system is fully automated and driven by a custom software written in Visual Basic that runs under the Metamorph software (Fig. S1C) allowing both piloting of the system and image processing.

### Reversible injections in the flow chamber system

**Figure 2:**
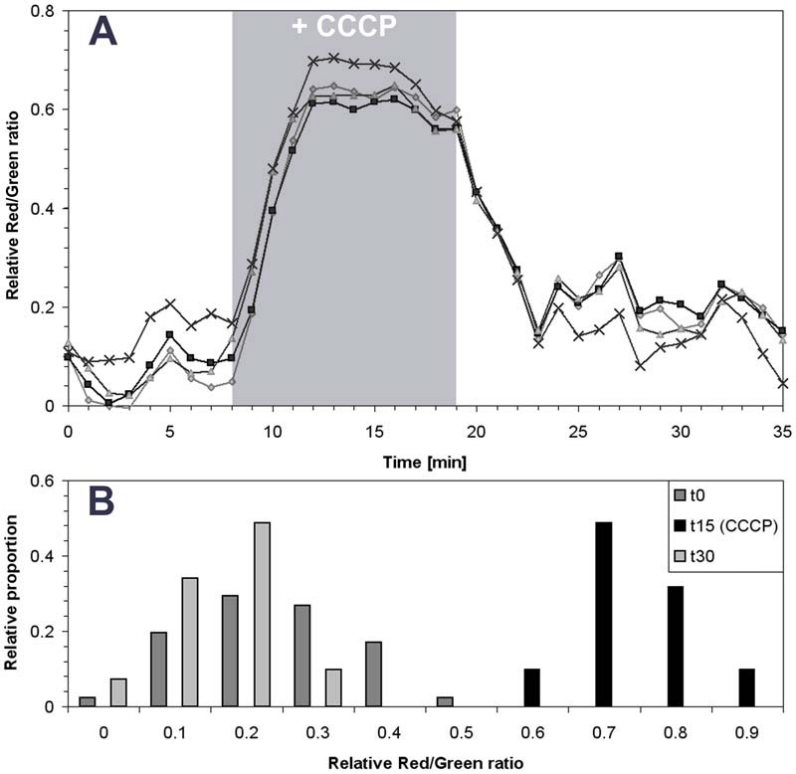
Reversible injections in the flow chamber system. A) Quantification of the membrane potential by monitoring the relative red/green fluorescence ratio after CCCP injection (indicated as grey shade) in cells stained by DiOC2. Each colored curve represents the kinetics of expression of a given single cell; B) Histogram of the relative red/green fluorescence ratio measured in 100 cells before (t0), during (t15) and after CCCP injection (t30).

A potential difficulty with the flow chamber system is the slow diffusion of molecules throught the agar pad, which could greatly limit its range of applications. To determine whether the chamber is suitable for real time studies of induced chemical perturbations, we tested the effect of the respiration uncoupler Carbonyl Cyanide m-Chloro Phenyl hydrazone (CCCP) which collapses the membrane potential of mitochondria and bacteria such as the gram negative bacterium *Myxococcus xanthus* (M. Wartel *et al*., unpublished). For this, *M. xanthus* cells were labelled with the fluorescent dye DiOC2 and placed inside the chamber system. DiOC2 is routinely used to measure perturbations in the membrane potential because its fluorescence switches from green to red emission when the membrane is depolarized [24], [25]. As shown in Fig. 2A, injection of CCCP (10 µM) rapidly shifted emission of DiOC2 to red fluorescence, showing that the drug reaches the cells in spite of the agar layer. Changes in the Red/Green fluorescence ratio were very synchronous across all cells and immediately observable, reaching a plateau ≈5 minutes after the beginning of injection (Fig. 2A). Such lag time is expected because at least 2 min are necessary to equilibrate the chamber with the CCCP solution when injected at 10 µl.s^−1^ and CCCP needs to diffuse to the cells through the agar. By comparison, when a similar experiment was conducted on bacteria fixed on poly-L-lysine and thus in direct contact with the flow, DiOC2 fluorescence emission changes reached a maximum after 2–3 min (data not shown), showing that CCCP diffuses rapidly and effectively through the agar pad.

The main advantage of a flow chamber system is that it allows to test whether the effect of given chemical is reversible. To test whether such studies may be conducted in our system, we flushed CCCP with medium (TPM) and monitored changes in red/green fluorescence over time (Fig. 2A). As expected, red/green fluorescence decreased rapidly, reaching values comparable to the pre-CCCP values ≈5 min after the beginning of TPM injection. Thus, CCCP effects and recovery are observed with similar kinetics strongly suggesting that CCCP does not interact with the agar pad and that the observed lag times are solely due to flow and diffusion requirements. Analysis of CCCP effects over 100 cells shows a normal distribution of the relative fluorescence ratios before, during and after treatment, showing that the effect of CCCP is fully reversible in our flow chamber system (Fig. 2B).

### Testing the physiological response of cells containing aggregated proteins

Aging in macroscopic organisms is deduced from obvious phenotypic traits. However, it is now becoming increasingly evident that single cell organisms also age [5], [12]. Identifying and quantifying aging and associated mechanisms in single cells can be challenging and has mostly been inferred from bulk phase experiments. In *Escherichia coli* aging has been associated to protein aggregation and the accumulation of oxidative damage caused by metabolic derivatives such as reactive oxygen species (ROS) [26]–[32]. Using fluorescence microscopy, Lindner *et al*., have shown that occurrence of protein aggregates (visualized using the Inclusion Body Protein A, IbpA) is directly correlated with cellular aging in single cells [5], [12]. The sequence of events that occur during aging remains unclear and it will be very interesting to study specific physiological responses in cells that contain protein aggregates. A flow chamber system is ideally suited to do such experiments: here, we tested in real time whether cells containing protein aggregates show altered responses to oxidative stress.

To test the response of cells containing aggregates to oxidative stress, we grew cells expressing both IbpA-YFP and a translational SodA-mCherry fusion (SodA is a Mn Superoxide dismutase) directly in the chamber and exposed the cells transiently to the oxydant Methyl Viologen (MV), well known to induce *sodA* [33]. *In vivo*, MV undergoes redox cycling so that it is first reduced by the electron donor NADPH, before it is oxidized again by an electron acceptor such as O_2_ to produce superoxide anions.

**Figure 3:**
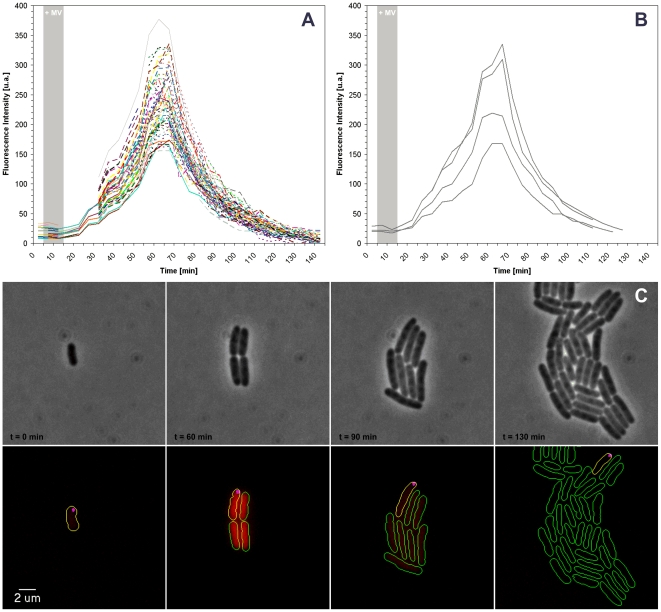
SodA-mcherry induction in cells containing inclusion bodies during MV injection. A) Measurement of SodA induction after Methyl Viologen injection and removal (indicated as grey shade) in cells carrying the SodA-mcherry fusion. Each colored curve represents the kinetics of expression of a given single cell B) Measurement of SodA induction in cells that carry polar foci of the protein aggregation marker IbpA-YFP. C) Sequence of images showing several rounds of cell division from a single mother cell carrying a polar IbpA-YFP polar focus; Top: phase images. Bottom: overlay of YFP (magenta) and mCherry fluorescent signals. Cell contours retrieved by the annotation software are added for clarity (yellow cells containing IbpA-YFP clusters and green cells not containing the clusters).

Inside the chamber, *E. coli* cells grew normally with doubling times comparable to those measured in liquid assays (Fig. 3C, Movie S1).At the beginning of the experiment about 50% of the cells contained at least one IbpA-YFP fluorescent foci at cellular poles, as previously described [12]. The IbpA-YFP foci rarely moved from their initial position so that, upon cell division, the cluster was only inherited by one of the daughter cells [12]. Interestingly, IbpA-YFP clusters were not newly formed in the course of the experiment (Fig. 3C, Movie S1). Injection of MV, led to very synchronous induction of SodA in all cells after a lag time of 7 min (Fig. 3A). To obtain reliable and quantitative measurements of SodA induction in cells with or without protein aggregates, we developed a script allowing automated and selective analysis of cells containing IbpA-YFP foci or not (see Methods). In cells containing IbpA-YFP the response to MV was indistinguishable from cells that contained no aggregates.

Thus, this experiment shows that cells containing protein aggregates still induce SodA when exposed to superoxide anions suggesting that protein aggregates do not impair the response to oxidative stress. This example shows that the chamber system is appropriate to study the fate and properties of specific cell sup-populations.

### Studies of bacterial motility: Myxococcus cells response to isoamyl-alcohol

Flow chamber systems are very useful tools to study dynamic processes such as cell motility. In eukaryotic cell systems, flow chamber assays have been used to study chemotaxis, energetics and cellular processes driving motility [17]–[20], [34]. By comparison, flow chamber approaches have only rarely been used to study bacterial motility at the single cell level. Studies in *Mycoplasma* used a flow chamber system to study the energetics of surface motility [35]. However, the substrate was specifically designed for *Mycoplasma* and is unlikely to be adaptable to other motile bacteria. In fact, a difficulty with flow chamber assays for the study of bacterial motility is the design of a substrate providing both a good level of adherence to resist the flow and allowing motility at the same time. In eukaryotes, proteins that mimick the extracellular matrix such as collagen and fibronectins have been widely used, but most bacteria that can perform surface motility have been studied on agar and do not move on these protein substrates. Finally, designing novel substrates for bacterial motility can be labour intensive and the results may not be directly comparable to measurements performed with agar assays. Thus, we decided to test whether our chamber system would allow studies of bacterial motility at the single cell level.

**Figure 4:**
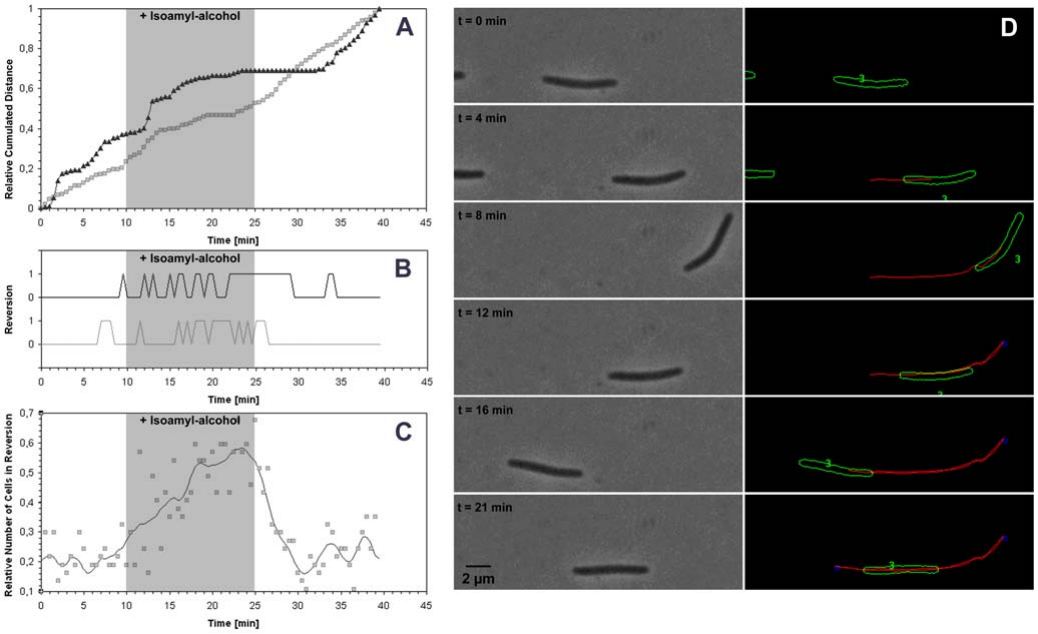
Response of Myxococcus cells to isoamyl-alcohol. A) Relative cel displacement during isoamyl-alcohol injection as a function of time (indicated as grey shade). Each curve represents a single cell. B) Occurrence of cell reversals during isoamyl-alcohol injection as a function of time (indicated as grey shade). Each curve shows the reversals of the cells shown in A, in black and in grey respectively. C) Relative proportion of cells undergoing reversals during isoamyl-alcohol injection as a function of time (indicated as grey shade) (n = 100) D) Left: Phase contrast images and automated scoring of a a single moving cell undergoing two reversals in the course of a 21 min time lapse. Right: cell contours with cell number (green), cell track (red) and scored reversals (blue square) as generated by the annotation software.

Movement of *Myxococcus* cells across solid surfaces allows this bacterium to perform a wide range of biological processes such as colony expansion, predation and fruiting body formation [36]. Rod-shaped *Myxococcus* cells change their direction of movement in a process termed cellular reversals where the initial leading pole is abruptly switched into the lagging pole, which results in movement in the opposite direction (Fig. 4D & Movie S2). Directional motility is achieved through the regulation of the frequency of reversals by a signal transduction pathway termed Frz [37], [38]. This way, *Myxococcus* cells can move up or down gradients of chemo-attractants/repellents in an Frz-dependent process. The response to isoamylalcohol (IA) has been studied in details as a *Myxococcus* repellent response, in presence of IA colony expansion is prevented due to IA-induced methylation of the Frz receptor [38]; *frz*-null mutants cannot sense IA and as a result colony expand despite the presence of the repellent (Fig. 9 in [37]). Analysis on moving Myxococcus cells on agar containing IA showed that the the cells reversed so frequently that they show no absolute net movement [38]. To test whether IA induces *frz*-dependent reversals directly, we exposed moving *Myxococcus* cells to IA transiently in our flow chamber system and scored the reversal frequency of the cells throughout treatment. To obtain reliable and quantitative measurements of cell velocities and reversal frequencies, we developed a script allowing automated analysis of our time-lapses (see Methods, Fig. 4D & Movie S2). Inside the chamber, *Myxococcus* cells moved normally with velocities comparable to those measured in standard agar assays (Fig. 4A and [39]). IA injection did not significantly alter overall cell velocity but greatly enhanced the reversal frequency (Fig. 4 A, B &C). As expected, removal of IA from the chamber rapidly restored the reversal frequencies to values comparable to the pre-IA values. In a similar control experiment, hyper-reversals where not observed when IA was injected to a *frz*-null mutant (data not shown). Thus, this experiment shows that IA directly induces hyper-reversals of *Myxococcus* cells. The chamber system is thus suitable to study chemical perturbations during bacterial motility.

## Discussion

We developed a hybrid micro fluidic device that combines a standard microbiology substratum (agar) with a custom flow chamber. We show that this setup is highly versatile because it supports growth, starvation and surface motility. The chamber is connected to a flow network that allows sequential injections of up to 12 different solutions. Automated injections are run through an user-friendly interface allowing simple piloting of the system.

The main advantage of our flow chamber is its high versatility and minimal montage requirements. Microscale flow networks have been largely used in eukaryotic cell biology [17]–[20] but it is a costly and demanding nanotechnology involving complex lithography for bacterial cells [3], [22]. Bacterial cell attachment to the chamber is also limiting because specific substrata must be designed to resist flows [23]. A hybrid microfluidic flow cell was recently developed by Charvin *et al*. In this system, a diffusive cellulose membrane separated the growing cells from the main chamber where the liquid was applied. *Saccharomyces cerevisiae* cells were clamped between a polydimethylsiloxane (PDMS) coated coverslip and the cellulose membrane, which mechanically constrained the cells to bud horizontally [11]. Charvin *et al*. could thus follow growing cells for more than 8 generations (<12 hrs) starting from single cells, with a controlled flow of the growth medium [11]. In principle, our system is similar to that of Charvin *et al*. but the use of an agar substratum makes it applicable to a broader range of studies. Agar pads are used in most laboratories that study bacterial physiology and cell biology [5], [12], [15], [16]. In some cases, an agar pad is the only substratum on which bacterial species can grow or move in the laboratory. Thus, this flow chamber system is amenable to most microbiology laboratories with an interest in using microfluidics to study bacterial physiology.

In classical microscopy applications, agar pads are sandwiched directly between the slide and the coverslip. Two major technical limitations may be identified: (i) desiccation may occur and lead to stress, hampering studies over the course of several hours and (ii) it is impossible in this way to change the growth conditions by switching the medium composition and study the reversible effect of drugs. In our system, the agar pad is constantly immersed in the culture medium, thus desiccation cannot occur. Moreover, medium may be injected at a constant flow for hours which may reduce potential stress from toxic metabolic compounds. One potential difficulty with our system is the slow diffusion of molecules through the agar pad, which could limit the range of applications. To test this potential limit, we used a 0.5 mm layer of agar and tested three molecules (CCCP, isoamyl-alcohol, and Methyl Viologen) in brief injections to test the biological response of bacteria clamped under the agar pad. In all cases, response occurred ≈3 min after injection and was fully reversible when the molecules were washed away with medium. Thus, the molecules tested do not interact with the agar pad and the observed lag times are solely due to flow and diffusion rates, suggesting that the pad is not a major diffusion barrier. Even if the system precludes observation of immediate effects, the measured 3 minutes lag time is compatible with the timescales of most biological events of interest.

We tested if the chamber would be appropriate to study cell fate after several generations in *E. coli*. Using a standard agar pad technique, Lindner *et al*. (2008) followed divisions of individual cells of *E. coli* cells through several lineages, and observed that the occurrence of protein aggregates could be correlated with cellular aging [5], [12]. As in all agar pad techniques a limitation is that cells tend to pile up after several generations, in our assays we could monitor successfully up to six generations. More development would be needed to follow cell lineages for longer times. Nevertheless our methodology allows us to reproduce the results of Lindner *et al*., but it also has the potential to increase the scope of the study because it offers the possibility to change the growth conditions in the course of the experiment. For instance, we show that cells containing the protein aggregates still induce *sodA* in a manner comparable to cells containing no detectable protein aggregates upon oxidative stress, indicating that the ability to cope against superoxide anions is not affected by the presence of protein aggregates. Thus, this approach allows to monitor the fates and behaviours of individual cells through multiple lineages.

The flow chamber system is useful to address questions were difficult to be address in absence of a specifically designed substratum. For example, movement of *Myxococcus xanthus* on agar is regulated by repellent molecules such as isoamylalcohol (IA). It has been proposed that IA-induced methylation of the Frz receptor leads to a state of “hyper-reversal” where the cells reverse so frequently that they show no absolute net movement, thus leading to the absence of colony expansion [37], [38]. However, this hypothesis could never be tested directly because cells readily resuspend when agar is overlaid in liquid. Here we directly tested the effect of IA on *Myxococcus* motility and observed that as expected, IA injection did not significantly alter the overall cell velocity whereas it greatly enhanced the reversal frequency. Our flow chamber system is thus adequate to directly study the effect of chemical perturbations on cell motility, which opens perspectives to study the mechanism of motility but also, on a larger more complex scale the dynamics of thousands of motile cells during biofilm formation.

In summary, we believe that the flow chamber system that we have developed will be very useful to study bacterial processes in real-time. The versatility of the system is such that it may be virtually adapted to study any processes that take place on an agar surface, whether it is a single round of cell division, the erection of a *Streptomyces* aerial hyphae or the formation of a complex bacterial community. Importantly, studies on the formation of bacterial micro-colonies [40] could now can be re-examined in a context of a much more controlled and less stressful environment.

## Materials and Methods

### Bacterial strains and growth

*M. xanthus* strains were grown at 32°C in CYE rich media which contains 10 mM MOPS pH 7.6, 1% (w/v) Bacto Casitone (BD Biosciences), 0.5% Bacto yeast extract and 4 mM MgSO_4_ [41].

*E. coli* strains. The *E. coli* (MG1655) endogenous *sodA* gene was replaced by homologous recombination by a chimeric gene encoding a SodA-mCherry translational fusion. *sodA-mcherry* was then introduced into the into the MGAY strain (kindly provided by A. Lindner [12]) expressing IbpA-YFP by P1 transduction. The mode of construction of strains and plasmids, as well as the sequences of all primers, are available upon request. *E. coli* cells were grown aerobically in liquid Luria-Bertani (LB) medium in a rotary shaker at 37°C and 160 rpm.

### Injection experiments

#### *M. xanthus* experiments

10 µL of a 4×10^8^ cfu/mL vegetative *M. xanthus* CYE cultures were spotted under a thin fresh TPM agar [42] and left to dry gently to absorb the cells onto the agar substrate (25 min). For injections, CCCP (10 µM) and isoamyl-alcohol (IA) were diluted to 10 µM and 0,3% in TPM medium containing Glucose (10 mM) respectively. CCCP and IA were removed from the chamber by injecting TPM containing Glucose (10 mM). For the CCCP experiments, TPM was also supplemented with the membrane potential marker DiOC2 according to the manufacturer's instructions (Invitrogen).

#### *E. coli* experiments

1 µL of a 1×10^7^ cfu/mL exponentially growing *E. coli* suspension cultures was spotted under a thin fresh agar as described above. The agar pad itself is only phosphate buffer-based, all necessary nutrients are provided by the flow (LB). Methyl viologen (MV) was used at 10 µM in LB medium. Subsequently, MV was removed by injecting LB.

### Microfluidic system and Microscopy

The microscope is placed within an incubator that allows precise regulation of the external temperature. The complete flow system consists of a rectangular flow chamber directly connected to custom designed electronic injectors. The flow chamber itself consists of a custom removable transparent lid sealed atop a standard coverslip by a parafilm rubber gasket. The cover slip is thus at the bottom of chamber and allows observation with an inverted microscope (Nikon TE2000-E-PFS, France). The chamber is maintained by a stainless steel base, which ensures sealing and system stability on the motorized stage of the microscope (Prior Scientific). The chamber is 20 mm long for a width of 10 mm and a depth of 1 mm, for a total volume of 200 µL. To avoid detachment of the cells by the flow and obtain micro-colonies to develop in a single plane from an individual cell, cells were inoculated directly on the cover slip and overlaid by a thin agarose pad. The thickness of the agarose pad was 0.5 mm and calibrated with the help of an agar pad maker device. The agar pad was left to dry gently at the borders which led to its absorption onto the coverslip, this proved critical for resistance to applied liquid flows. At the top of the chamber, are two luer systems allowing connection to the injectors. Syringe pumps were used in aspiration mode and were specifically designed to ensure a regular flow velocity and avoid perturbation of the specimen focus. Used flow velocities ranged between 0,1 µL/s and 100 µL/s, were controlled by the injector pump and were calibrated by measuring flow rates at various pump speeds.

The fluidic network system itself comprises 12 valves especially designed to have a very limited void volumes thus allowing sequential or parallel injection of up to 12 distinct solutions. Potential air bubbles in the tubing were eliminated by coupling the downstream valve to a bubble detector system.

### Data Acquisition

Microscopic analysis was performed using an automated and inverted epifluorescence microscope TE2000-E-PFS (Nikon, France). The microscope is equipped with “The Perfect Focus System” (PFS) that automatically maintains focus so that the point of interest within a specimen is always kept in sharp focus at all times, in spite of any mechanical or thermal perturbations.

For each experiment, ten separate fields containing at least 20 cells each, were manually or automatically defined with a motorized stage (Prior Scientific) and stored (X, Y, Z, PFS-offset) in our custom automation system designed for time-lapse experiments. Images were recorded with a CoolSNAP HQ 2 (Roper Scientific, Roper Scientific SARL, France) and a 40x/0.75 DLL “Plan-Apochromat” or a 100x/1.4 DLL objective. Resulting images have spatial dimensions of 0.16 µm/pixel and 0.064 µm/pixel, respectively. Excitation light was emitted by a 120 W metal halide light and signals were monitored using appropriate filters. All fluorescence images were acquired with a minimal exposure time to minimize bleaching and phototoxicity effects. As a control, a field that was not exposed to fluorescent illumination was monitored in similar conditions. In our conditions, the growth or the motility of cells were not affected by the illumination (Data not shown).

Time-lapse experiments, digital analysis and image processing were conduced by a custom automation script (Visual Basic) under Metamorph 7.5 (Molecular Devices, Molecular Devices France, France).

### Data Analysis

Prior to any image processing, phase-contrast or fluorescent gray-scale images used for segmentation were equalized to reduce background noise fluctuations. Cells were then automatically segmented by successive morphological operation involving h-dome extraction, gray-scale reconstruction [43], [44], binary images, and morphological opening. To optimize segmentation, binary frames were sometimes manually corrected with appropriate tools built into the software. A binary mask was then used to perform integrated morphometric analysis and cell tracking. Cell tracking was performed following standard mathematical procedures [45], [46]. When appropriate, manual measurements were also performed to correct tracking errors generated by the software. The morphometric data were processed under Excel (Microsoft).

## Supporting Information

Figure S1 Overall principle of the setup. A) The hybrid flow chamber. B) Diagram of the automated flow networks. The 12 separate valves are connected to a bubble detector (BS) that isolates the flow chamber from potential bubbles in the flow network by triggering two additional valves. Aspiration of the different solutions is accomplished by a syringe pump (SP) connected to the waste (W). C) Overall principle of the setup. The whole setup is driven by a custom-made Visual Basic application run on a PC computer, through multiple RS232 interface (except the camera which has FireWire interface). The microscope (1), the control unit for the electric valves (2), the syringe pump (3), the camera (4), and the motorized stage can be remotely controlled by our custom program.
(0.84 MB TIF)

Movie S1 Transient SodA:mcherry expression among cell division. Left: Sequence of images showing an overlay of phase contrast and YFP fluorescence of a colony from a cell that carry a foci of the protein aggregation marker ibpA-YFP; right: Sequence of images showing an overlay of mcherry fluorescence and cell contours as retrieved by the annotation software. Cell contour of cell containing identified foci of the protein aggregation marker ibpA-YFP (magenta) are in yellow and contour of other cells are in green. Each frame was taking every 5 min.
(0.95 MB MOV)

Movie S2 Tracking of reversion events of a moving cell. Up: Sequence of images showing phase contrast of a single moving cell that carry two reversion during 21 min; down: cell contours with cell number (green), cell's track (red) and reversion (blue square) as retrieved by the annotation software. Each frame was taking every 30 sec.
(1.89 MB MOV)
