# Supplementary figures and images for: A Microscope Automated Fluidic System to Study Bacterial Processes in Real Time
Source: PLoS One. 2009 Sep 30;4(9):e7282. doi: 10.1371/journal.pone.0007282 (PMC2748647; doi:10.1371/journal.pone.0007282)

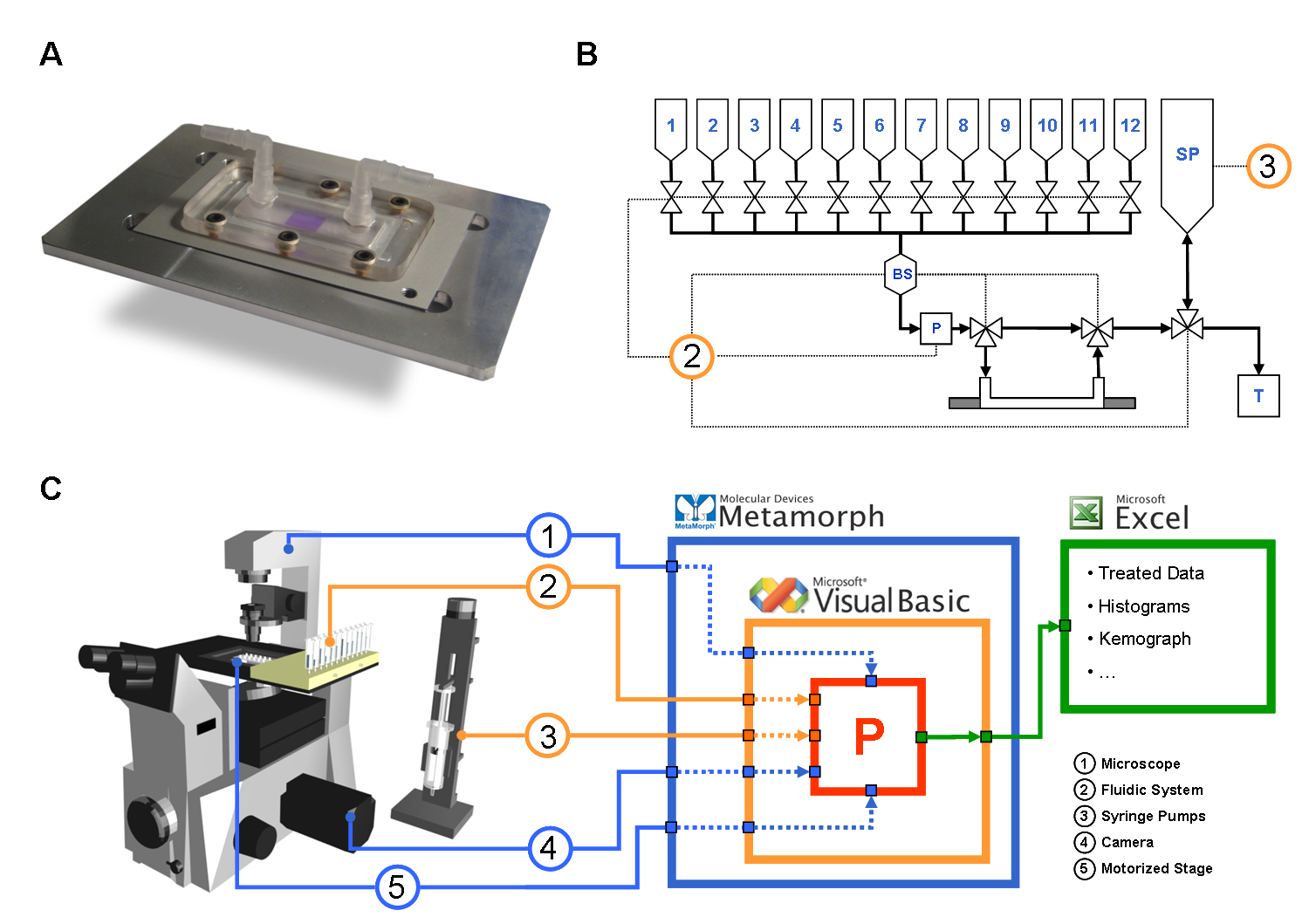

Supplement: Figure S1 — Overall principle of the setup. A) The hybrid flow chamber. B) Diagram of the automated flow networks. The 12 separate valves are connected to a bubble detector (BS) that isolates the flow chamber from potential bubbles in the flow network by triggering two additional valves. Aspiration of the different solutions is accomplished by a syringe pump (SP) connected to the waste (W). C) Overall principle of the setup. The whole setup is driven by a custom-made Visual Basic application run on a PC computer, through multiple RS232 interface (except the camera which has FireWire interface). The microscope (1), the control unit for the electric valves (2), the syringe pump (3), the camera (4), and the motorized stage can be remotely controlled by our custom program. (0.84 MB TIF) [file pone.0007282.s001.tif]
